# Correction: A functional interleukin-4 homolog is encoded in the genome of infectious laryngotracheitis virus: Unveiling a novel virulence factor

**DOI:** 10.1371/journal.ppat.1013791

**Published:** 2025-12-19

**Authors:** Jeremy D. Volkening, Stephen J. Spatz, Maricarmen Garcia, Teresa A. Ross, Daniel A. Maekawa, Kenneth S. Rosenthal, Ana C. Zamora, April Skipper, Julia R. Blakey, Rashan Paudel

The tenth author’s name is spelled incorrectly. The correct name is: Roshan Paudel.
