# Supplementary material for: A functional interleukin-4 homolog is encoded in the genome of infectious laryngotracheitis virus: Unveiling a novel virulence factor
Source: PLoS Pathog. 2025 Jul 23;21(7):e1013219. doi: 10.1371/journal.ppat.1013219 (PMC12327624; doi:10.1371/journal.ppat.1013219)
Supplement: S1 Table — Accessions are from GenBank and are either the direct protein entries used or the genomic sequences on which the predicted genes are located (as indicated in the Notes). (PDF) [file ppat.1013219.s001.pdf]

Table S1: Vertebrate IL-4 homologs used in the analysis. Accessions are from GenBank and are either the direct protein entries used or the genomic sequences on which the predicted genes are located (as indicated in the Notes).

Common name	Scientific name	Accession	Notes
vIL-4	Gallid alpha-herpesvirus 1	N/A	from GaHV-1 1874C5 IsoSeq and internal 1874C5 corrected genome
chicken	Gallus gallus	NM_001398459.1	used as-is
Chinese bamboo-partridge	Bambusicola thoracicus	POI22934.1	miniprot/AUGUSTUS prediction, matches indicated protein accession
turkey	Meleagris gallopavo	ENSMGAT00000023360.1	GenBank NM_001303181.1 is actually IL13
ring-necked pheasant	Phasianus colchicus	NW_022205427.1	miniprot/AUGUSTUS prediction
greater sage-grouse	Centrocercus urophasianus	JAHKSY010000026.1	miniprot/AUGUSTUS prediction
rock ptarmigan	Lagopus muta	CM041489.1	miniprot/AUGUSTUS prediction
green peafowl	Pavo muticus	JACDJE010000203.1	miniprot/AUGUSTUS prediction
red-legged partridge	Alectoris rufa	CAMZON010000379.1	miniprot/AUGUSTUS prediction
Japanese quail	Coturnix japonica	XM_015875546.1	used as-is
guineafowl	Numida meleagris	XM_021410541.1	used as-is
guan	Penelope pileata	WBMW01000786.1	miniprot/AUGUSTUS prediction
bobwhite	Colinus virginianus	AWGT02000115.1	miniprot/AUGUSTUS, structure differs from OXB76676
brush-turkey	Alectura lathami	VXAV01004783.1	miniprot/AUGUSTUS prediction
tufted duck	Aythya fuligula	XM_032196612.1	used as-is
mallard	Anas platyrhynchos	NC_051785.1	miniprot/AUGUSTUS, several nucleotide positions differ from MF346730.1
black swan	Cygnus atratus	XM_035560814.1	used as-is

Table S1: Vertebrate IL-4 homologs used in the analysis. Accessions are from GenBank *(continued)*

Common name	Scientific name	Accession	Notes
egret	Egretta garzetta	XM_009647167.1	used as-is
Adelie penguin	Pygoscelis adeliae	XM_009322783.1	used as-is
goshawk	Accipiter gentilis	XM_049829109.1	used as-is
golden eagle	Aquila chrysaetos	XM_029998407.1	used as-is
zebra finch	Taeniopygia guttata	NC_044225.2	miniprot/AUGUSTUS, structure differs from XM_041718987.1
song sparrow	Melospiza melodia maxima	RZID01002771.1	used as-is
ostrich	Struthio camelus	JJRT01005166.1	miniprot/AUGUSTUS, structure differs from XM_009679367.1
emu	Dromaius novaehollandiae	NW_020453704.1	miniprot/AUGUSTUS, structure differs from XP_025974110.1
tinamou	Nothoprocta perdicaria	NW_020454172.1	miniprot/AUGUSTUS, structure differs from XM_026045620.1
saltwater crocodile	Crocodylus porosus	NW_017728914.1	miniprot/AUGUSTUS prediction
alligator	Alligator sinensis	XM_006023567.1	used as-is
slider turtle	Trachemys scripta elegans	NC_048305.1	miniprot/AUGUSTUS, structure differs from XM_03477890.1
green sea turtle	Chelonia mydas	NC_057854.1	miniprot/AUGUSTUS prediction
