# Supplementary material for: A functional interleukin-4 homolog is encoded in the genome of infectious laryngotracheitis virus: Unveiling a novel virulence factor
Source: PLoS Pathog. 2025 Jul 23;21(7):e1013219. doi: 10.1371/journal.ppat.1013219 (PMC12327624; doi:10.1371/journal.ppat.1013219)
Supplement: S2 Table — Columns shown are: peptide sequence with post-translation modification (PTM) masses in square brackets; minimum q-value assigned (from all samples); maximum per-sample PSM count observed; observed charge states across all samples; replicates the peptide proteoform was detected in; search engines identifying the specific proteoform. Note that where PTMs could not be confidently localized to a single amino acid, different search engines may prefer and report different modified positions. An example is the deamidated form of peptide LQGLYVNLYYVLNSVSNGGSSRK. (PDF) [file ppat.1013219.s002.pdf]

Table S2: All vIL-4 peptide proteoforms detected by LC-MS/MS of infected cell culture at an FDR of 1%. Columns shown are: peptide sequence with post-translation modification (PTM) masses in square brackets; minimum q-value assigned (from all samples); maximum per-sample PSM count observed; observed charge states across all samples; replicates the peptide proteoform was detected in; search engines identifying the specific proteoform. Note that where PTMs could not be confidently localized to a single amino acid, different search engines may prefer and report different modified positions. An example is the deamidated form of peptide LQGLYVNLYYVLNSVSNGGSSRK.

peptide	minimum q-value	max # PSMs	charge states	found in samples	found by engines
APCPITVEDSYLM[+16]K	0.0000	10	+2,+3	1874C5-A,1874C5-B,1874C5-C,LV-A,LV-B,LV-C	Comet,MS-GF+
APCPITVEDSYLMK	0.0000	3	+2	1874C5-C,LV-A,LV-C	Comet,MS-GF+
KAPCPITVEDSYLM[+16]K	0.0000	8	+3,+2	1874C5-B,1874C5-C,LV-A,LV-B,LV-C	Comet,MS-GF+
KAPCPITVEDSYLMK	0.0000	6	+3,+2	1874C5-A,LV-A,LV-B,LV-C	Comet,MS-GF+
KLQGLYVNLYYVLNSVSN[+1]GGSSR	0.0001	7	+3	1874C5-A,1874C5-B,1874C5-C,LV-A,LV-B,LV-C	Comet
KLQGLYVNLYYVLNSVSN[+1]GGSSRK	0.0001	8	+3,+4	1874C5-A,LV-A,LV-B,LV-C	Comet
KLQGLYVNLYYVLNSVSNGGSSR	0.0000	6	+3	1874C5-A,1874C5-C,LV-A,LV-B,LV-C	Comet,MS-GF+
LLAESIR	0.0005	10	+2,+1	1874C5-A,1874C5-C,LV-A,LV-B,LV-C	Comet,MS-GF+
LNETEELCQSLSVILK	0.0000	15	+2,+3	1874C5-A,1874C5-B,1874C5-C,LV-A,LV-B,LV-C	Comet,MS-GF+
LQGLYVNLYYVLN[+1]SVSNGGSSRK	0.0008	3	+3	LV-A,LV-B,LV-C	MS-GF+
LQGLYVNLYYVLNSVSN[+1]GGSSR	0.0001	12	+3,+2	1874C5-A,1874C5-B,1874C5-C,LV-A,LV-B,LV-C	Comet
LQGLYVNLYYVLNSVSN[+1]GGSSRK	0.0007	2	+3	LV-B,LV-C	Comet
LQGLYVNLYYVLNSVSNGGSSR	0.0000	3	+3	LV-A,LV-B,LV-C	Comet,MS-GF+
LQGLYVNLYYVLNSVSNGGSSRK	0.0000	3	+3	LV-A,LV-B,LV-C	Comet,MS-GF+
RVPNIFTSDTR	0.0000	11	+2,+3	1874C5-A,1874C5-B,1874C5-C,LV-A,LV-B,LV-C	Comet,MS-GF+
RVPNIFTSDTRLNETEELCQSLSVILK	0.0000	2	+4	LV-A,LV-B	Comet,MS-GF+
SFLNDLAN[+1]ALQR	0.0000	7	+2	1874C5-B,1874C5-C,LV-A,LV-B,LV-C	Comet,MS-GF+
SFLNDLANALQ[+1]R	0.0033	1	+2	1874C5-B	Comet
SFLNDLANALQR	0.0000	21	+2,+3	1874C5-A,1874C5-B,1874C5-C,LV-A,LV-B,LV-C	Comet,MS-GF+
SFLNDLANALQRR	0.0000	5	+3,+2	1874C5-A,LV-A,LV-B,LV-C	Comet,MS-GF+
VPNIFTSDTR	0.0000	1	+2	1874C5-C	Comet,MS-GF+
