# Supplementary material for: A functional interleukin-4 homolog is encoded in the genome of infectious laryngotracheitis virus: Unveiling a novel virulence factor
Source: PLoS Pathog. 2025 Jul 23;21(7):e1013219. doi: 10.1371/journal.ppat.1013219 (PMC12327624; doi:10.1371/journal.ppat.1013219)
Supplement: S1 Fig — (PDF) [file ppat.1013219.s003.pdf]

## Supplementary materials

| | | |
|---|---|---|
| **cIL4 Inserts** | | |
| VTRmuGFPFORC-IL4 | TCAGATCCGCTAGCGCCGCCATGCCAGGCAAGAGCAAGCC | Set 1 |
| muGFP C-IL4 REV | CACAGACAGCTGCAGACACAGTGTGAACTCTCCGCTTCCAGCTGC | |
| C-IL4 MuGFP For | GCAGCTGGAAGCGGAGAGTTCACACTGTGTCTGCAGCTGTCTGTG | Set2 |
| cIL4 Rev | TACCGTCGACTGCAGAATTCGAAGCTTGAGCTCGAGTCACTTGTTCTTGGCCAGC | |
| | | |
| **cIL4 vector** | | |
| vtr cIL4 for | CACCAGCTGGCCAAGAACAAGTGACTCGAGCTCAAGCTTCGAATTCTGCAGTC | Set 3 |
| vtr MuGFP REV C-IL4 | GGCTTGCTCTTGCCTGGCATGGCGGCGCTAGCGGATCTG | |
| | | |
| | | |
| **vIL4 Inserts** | | |
| VirIL4 For | CAGATCCGCTAGCAGACGCCACCATGCCAGGCAAGAGCAAGCCAC | Set 4 |
| muGFP vIL4 REV | TGGAGCGGCTCTAATGCTGCGGAACTCTCCGCTTCCAGCTGC | |
| vIL4 MuGFP For | GCAGCTGGAAGCGGAGAGTTCCGCAGCATTAGAGCCGCTCCA | Set 5 |
| Vir IL4 Rev | AGCTTGAGCTCGAGAGTTACATCAGTACTGCCTGCTCTGTCTGACGT | |
| | | |
| **vIL4 vector** | | |
| vtr Vir IL4 for | GATACGTCAGACAGAGCAGGCAGTACTGATGTAACTCTCGAGCTCAAGCT | Set 6 |
| vtr Vir IL4 rev | GTGGTGGCTTGCTCTTGCCTGGCATGGTGGCGTCTGCTAGCGGAT | |

Figure S1: List of primers used to generate PCR amplicons, which were assembled to create N-terminal tagged versions of cIL-4 and vIL-4.
