# Supplementary material for: A functional interleukin-4 homolog is encoded in the genome of infectious laryngotracheitis virus: Unveiling a novel virulence factor
Source: PLoS Pathog. 2025 Jul 23;21(7):e1013219. doi: 10.1371/journal.ppat.1013219 (PMC12327624; doi:10.1371/journal.ppat.1013219)
Supplement: S2 Fig — (A) Diagram of the plasmid constructs expressing GFP-tagged cIL-4 and vIL-4 proteins. Fluorescent microscopy of LMH cells transfected with (B) GFP-cIL-4 and (C) GFP-vIL4. Magnification is 100 × . (PDF) [file ppat.1013219.s004.pdf]

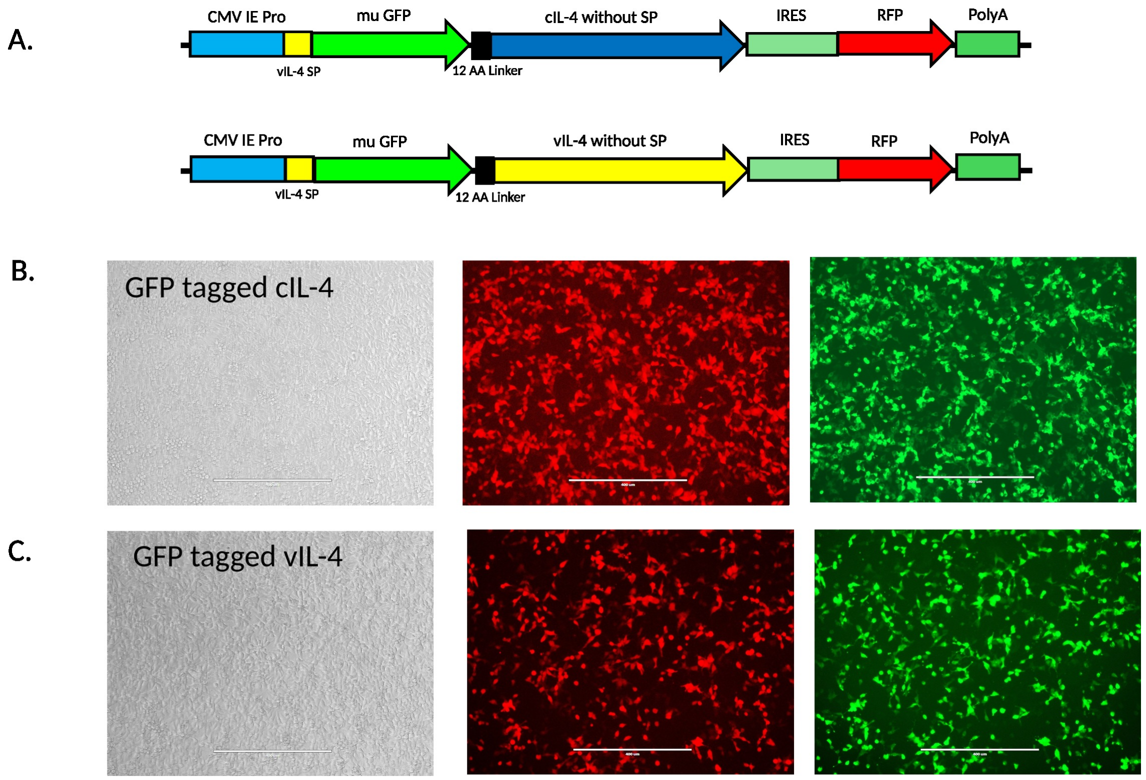

Figure S2: GFP-tagged cIL-4 and vIL-4. (A) Diagram of the plasmid constructs expressing GFP-tagged cIL-4 and vIL-4 proteins. Fluorescent microscopy of LMH cells transfected with (B) GFP-cIL-4 and (C) GFP-vIL4. Magnification is 100×.
