# Supplementary material for: A functional interleukin-4 homolog is encoded in the genome of infectious laryngotracheitis virus: Unveiling a novel virulence factor
Source: PLoS Pathog. 2025 Jul 23;21(7):e1013219. doi: 10.1371/journal.ppat.1013219 (PMC12327624; doi:10.1371/journal.ppat.1013219)
Supplement: S3 Fig — (A) Schematic of the ILTV D-type genome, showing the unique long (UL) and unique short (US) regions flanked by inverted repeats (internal repeat short [IRS] and terminal repeat short [TRS]). (B) Gene organization at the UL/TRS junction, indicating gene direction. (C) The vIL-4 locus, with exons (arrows connected by lines) and their corresponding coordinates based on GenBank entry JN542533: Exon 1 (114,156–114,302), Exon 2 (114,422–114,468), Exon 3 (114,629–114,768), and Exon 4 (114,919–115,028). White triangles indicate the guide RNA (gRNA) binding sites. Black triangles denote the locations of forward (F1-4, TAAGGCCGCAATTAGGTGCT; 113,787–113,806) and reverse (R1-4, AGCCCCGTTGACTTTAGCTT; 115,278–115,259) oligonucleotide primers used for amplicon generation. (D) Sanger sequencing data of an amplicon from plaque-purified ΔvIL-4 mutant (#17). Arrows denote the position of the deletion. (PDF) [file ppat.1013219.s005.pdf]

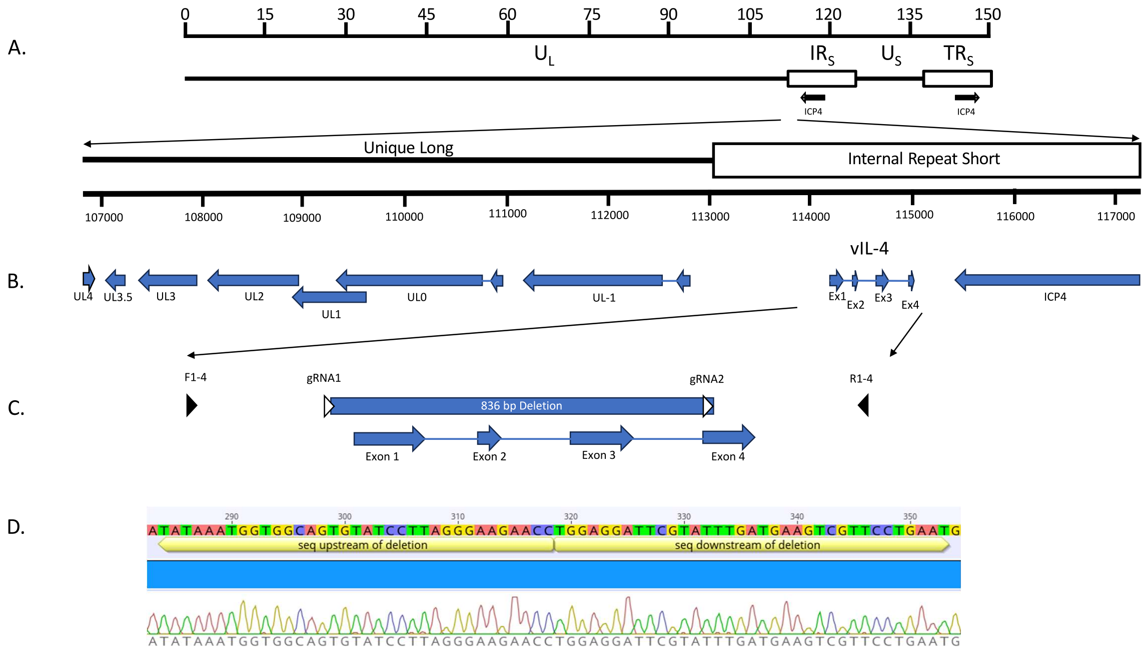

Figure S3: Generation of a vIL-4 deletion mutant. (A) Schematic of the ILTV D-type genome, showing the unique long (UL) and unique short (US) regions flanked by inverted repeats (internal repeat short [IRS] and terminal repeat short [TRS]). (B) Gene organization at the UL/TRS junction, indicating gene direction. (C) The vIL-4 locus, with exons (arrows connected by lines) and their corresponding coordinates based on GenBank entry JN542533: Exon 1 (114,156–114,302), Exon 2 (114,422–114,468), Exon 3 (114,629–114,768), and Exon 4 (114,919–115,028). White triangles indicate the guide RNA (gRNA) binding sites. Black triangles denote the locations of forward (F1-4, TAAGGCCGCAATTAGGTGCT; 113,787–113,806) and reverse (R1-4, AGCCCCGTTGACTTTAGCTT; 115,278–115,259) oligonucleotide primers used for amplicon generation. (D) Sanger sequencing data of an amplicon from plaque-purified ΔvIL-4 mutant (#17). Arrows denote the position of the deletion.
