# Supplementary material for: A functional interleukin-4 homolog is encoded in the genome of infectious laryngotracheitis virus: Unveiling a novel virulence factor
Source: PLoS Pathog. 2025 Jul 23;21(7):e1013219. doi: 10.1371/journal.ppat.1013219 (PMC12327624; doi:10.1371/journal.ppat.1013219)
Supplement: S4 Fig — (PDF) [file ppat.1013219.s006.pdf]

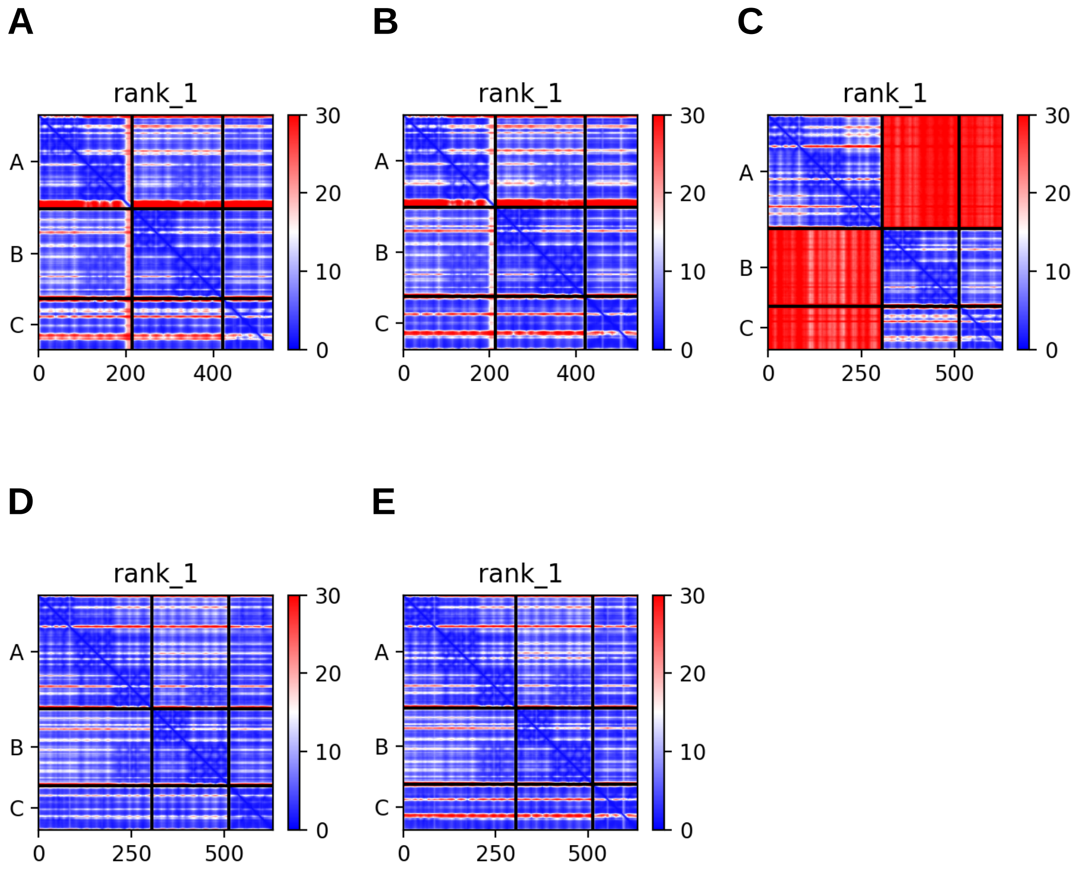

Figure S4: Predicted aligned error (PAE) plots of AlphaFold multimeric models for (A) gamma-c + IL-4Ra + cIL-4; (B) gamma-c + IL-4Ra + vIL-4; (C) IL-13Ra1 + IL-4Ra + cIL-4; (D) IL-13Ra1 + IL-4Ra + cIL-13; (E) IL-13Ra1 + IL-4Ra + vIL-4.
