# Supplementary material for: A functional interleukin-4 homolog is encoded in the genome of infectious laryngotracheitis virus: Unveiling a novel virulence factor
Source: PLoS Pathog. 2025 Jul 23;21(7):e1013219. doi: 10.1371/journal.ppat.1013219 (PMC12327624; doi:10.1371/journal.ppat.1013219)
Supplement: S5 Fig — The putative leader sequence has been removed. Red bars are identified peptides (1% FDR) including peptides with missed cleavages. Dark gray bars indicate theoretical tryptic peptides with GRAVY (GRand AVerage of hydropathY) score < 0.5. Light gray bars indicate theoretical tryptic peptides with GRAVY score >= 0.5 (more hydrophobic and thus possibly more difficult to detect). Tryptic peptides shorter than 6 aa were not searched and are not shown. (PDF) [file ppat.1013219.s007.pdf]

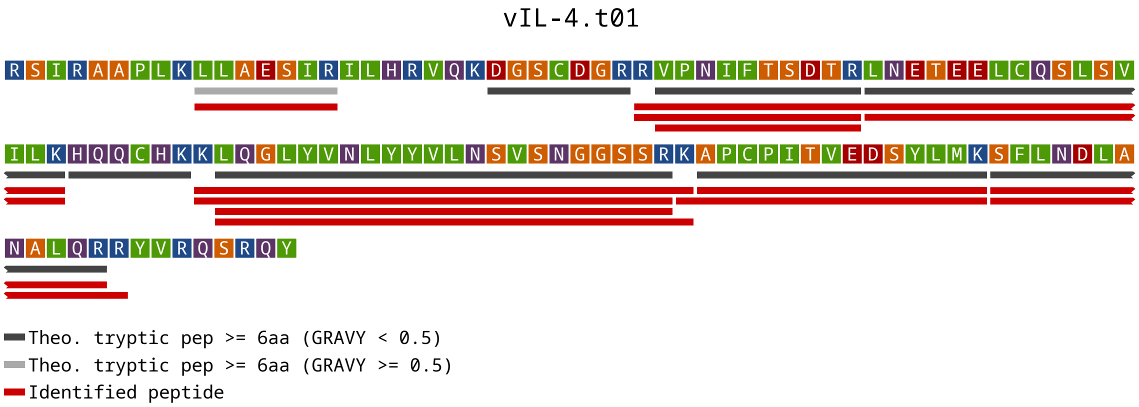

Figure S5: Map of identified and theoretical trypic peptides within the vIL-4 protein. The putative leader sequence has been removed. Red bars are identified peptides (1% FDR) including peptides with missed cleavages. Dark gray bars indicate theoretical tryptic peptides with GRAVY (GRand AVerage of hydropathY) score < 0.5. Light gray bars indicate theoretical tryptic peptides with GRAVY score >= 0.5 (more hydrophobic and thus possibly more difficult to detect). Tryptic peptides shorter than 6 aa were not searched and are not shown.
