# Supplementary material for: A functional interleukin-4 homolog is encoded in the genome of infectious laryngotracheitis virus: Unveiling a novel virulence factor
Source: PLoS Pathog. 2025 Jul 23;21(7):e1013219. doi: 10.1371/journal.ppat.1013219 (PMC12327624; doi:10.1371/journal.ppat.1013219)
Supplement: S7 Fig — Top rows show up to three MS2 spectra matched at 1% FDR, with matching y-b ion series labeled in blue and red. On the right of each row is the MS1 precursor window used for fragmentation. The bottom row shows an extracted ion chromatogram (XIC) for the peptide precursor mass over the matching retention time window for all nine samples (1875C5, Laryngo-Vac, and mock). True viral peptides should have XIC elution peaks in infected samples but not mock. (PDF) [file ppat.1013219.s009.pdf]

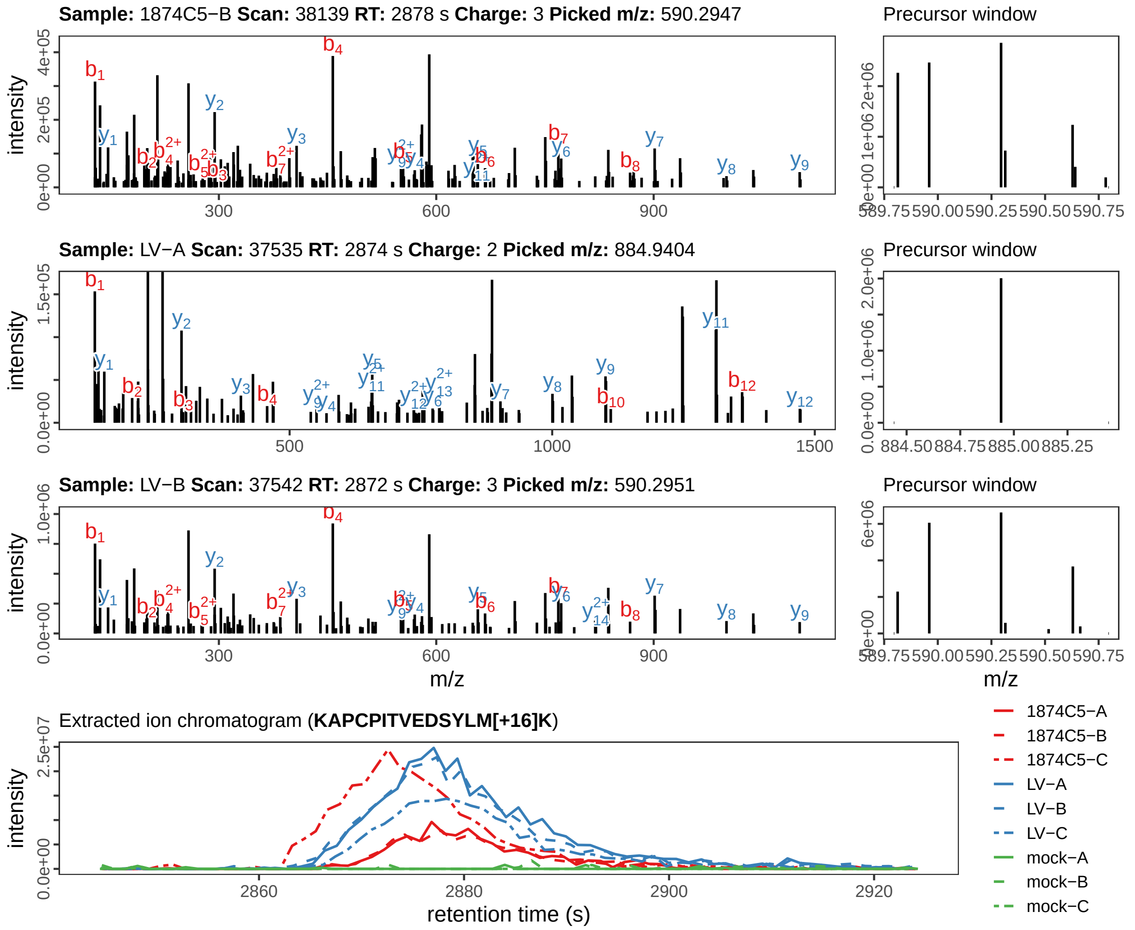

Figure S7: QC data for vIL-4 peptide KAPCPITVEDSYLM[+16]K identified by LC-MS/MS. Top rows show up to three MS2 spectra matched at 1% FDR, with matching y-b ion series labeled in blue and red. On the right of each row is the MS1 precursor window used for fragmentation. The bottom row shows an extracted ion chromatogram (XIC) for the peptide precursor mass over the matching retention time window for all nine samples (1875C5, LaryngoVac, and mock). True viral peptides should have XIC elution peaks in infected samples but not mock.
